# Approach to Neurological Channelopathies and Neurometabolic Disorders in Newborns

**DOI:** 10.3390/life11111244

**Published:** 2021-11-16

**Authors:** Inn-Chi Lee

**Affiliations:** 1 Division of Pediatric Neurology, Department of Pediatrics, Chung Shan Medical University Hospital, Taichung 40201, Taiwan; 2 Institute of Medicine, School of Medicine, Chung Shan Medical University, Taichung 40201, Taiwan

**Keywords:** channelopathies, neurometabolic, newborns, seizures

## Abstract

Ion channel disorders (channelopathies) can affect any organ system in newborns before 2 months of life, including the skeletal muscle and central nervous system. Channelopathies in newborns can manifest as seizure disorders, which is a critical issue as early onset seizures can mimic the presentation of neurometabolic disorders. Seizures in channelopathies can either be focal or generalized, and range in severity from benign to epileptic encephalopathies that may lead to developmental regression and eventually premature death. The presenting symptoms of channelopathies are challenging for clinicians to decipher, such that an extensive diagnostic survey through a precise step-by-step process is vital. Early diagnosis of a newborn’s disease, either as a channelopathy or neurometabolic disorder, is important for the long-term neurodevelopment of the child.

## 1. Introduction

Ion channel disorders (channelopathies) can affect any organ system in newborns before 2 months of age, but they mainly affect the skeletal muscle or central nervous system [1]. Channelopathy can involve the dysfunction of potassium, sodium, chloride, or calcium ion channels, as well as acetylcholine and glycine receptors. The incidence of encephalopathy due to channelopathies remains unknown. Neonatal neurological metabolic disorders can be caused by genetic defects and biochemical and molecular abnormalities. Most of these disorders are autosomal recessive or maternally inherited by an enzyme deficiency in the metabolic pathway. Neurological channelopathy genes implicated in the pathogenesis of newborns include *KCNQ2* (a voltage-gated potassium channel), *KCNQ3* (a voltage-gated potassium channel), *SCN2A* (a voltage-gated sodium channel Na(v)1.2), *SCN8A* (sodium voltage-gated channel alpha subunit 8), and *CACAN1A* (CaV2.1 voltage-gated calcium channel). Neurologic channelopathies can either manifest as a mild and benign epileptic syndrome with normal neurodevelopment or as severe symptoms, such as severe encephalopathy, refractory seizures, and other neurodevelopmental disorders, which are heterogeneous in etiologies, as channelopathies can be caused by more than 50 genes.

Neurometabolic diseases and channelopathies in newborns are differentiated by clinicians through observation of refractory seizures, changes in consciousness, feeding problems, and apnea patterns. Neurometabolic diseases are complex and heterogeneous. The electroencephalogram (EEG) of both neurometabolic diseases and channelopathies can have similar findings (suppression-burst (SB) EEG, multiple focal spike), making these two categories indistinguishable. Therefore, differentiating the presenting symptoms of channelopathies and neurometabolic disorders can be challenging for clinicians. An extensive diagnostic survey with a precise step-by-step process must be conducted. Early diagnosis, either as a channelopathy or neurometabolic disease, is critical to the long-term neurodevelopment of a child. The aim of this article is to help clinicians in reviewing potential metabolic disorders and neonatal epilepsy for approaching both disorders.

## 2. Neurological Disease in Newborns with Heterogenous Etiologies

In newborns, genetic disorders can cause severe neurological diseases, congenital malformations, inborn errors of metabolism, and developmental epileptic encephalopathy (DEE) [2,3,4,5,6]. Seizures usually occur early in life, particularly during infancy. A common etiology of neonatal-infantile seizures involves the mutation of the genes *KCNQ2*, *SCN1A*, and *SCN2A* [7,8,9,10,11,12,13]. The earliest onset of seizures could occur before 2 months of age [8,13]. Other etiologies include hypoxic ischemic encephalopathy (HIE), hypocalcemia (early- and late-onset), hypoglycemia, sepsis or meningitis, and neurological disorders of premature infants. Uncommon etiologies include vitamin B_6_ deficiency, hyperthyroidism, and metabolic disorders (i.e., urea cycle disorders, organic acidemia, maple syrup urine disease, and fatty acid oxidation disorders). It is crucial to consider a diagnosis of treatable HIE, meningoencephalitis or intracranial hemorrhage in the early stage; however, these conditions lack typical findings observed in older infants and children, and seizures may be the only early symptom. Channelopathies and neurometabolic disorders can manifest as seizures early in life, even immediately after birth [2,6,14,15]. Understanding these factors can avoid extensive diagnosis, thereby prompting initiation of treatment, and improving long-term outcomes.

### 2.1. Neurometabolic Disorders in Newborns

Urea cycle disorders, organic acidemia, maple syrup urine disease, and fatty acid oxidation disorders present with encephalopathy within a few days of life after a period of normalcy. Normal tandem mass spectrometry, plasma amino acids, and urine organic acids rule out these disorders [14,16,17]. Seizures are not the predominant manifestations. On the contrary, nonketotic hyperglycinemia, molybdenum cofactor deficiency, pyridoxal 5′-phosphate-dependent seizures, folinic acid-responsive seizures, and serine synthesis defects present with intractable seizures within a few hours to days after birth. Moreover, as biochemical investigations are not suggestive, these disorders are unlikely to be the aforementioned set of neurometabolic diseases [6,15,16,17].

### 2.2. Mitochondrial Disease

Mitochondrial dysfunction can occur in premature neonates, intrauterine growth restriction, and hypotonia with respiratory failure that often requires ventilation, cardiomyopathy, and encephalopathy. Elevated plasma lactate is a biochemical marker of mitochondrial dysfunction [18,19,20]. Early onset encephalopathy with tone abnormalities, elevated lactate in blood, and lactate peak in magnetic resonance spectroscopy (MRS) led to a possibility of mitochondrial disease [6,14,17].

### 2.3. Risks for the Patient When a Misdiagnosis Is Made between Channelopathy and a Metabolic Disorder

Neonatal channelopathy and neurometabolic disorder outcomes are determined by the genotype and acquired factors, such as duration of seizure cessation and environmental factors. In patients with a misdiagnosis, such as pyridoxine-dependent epilepsy, delayed treatment can result in secondary brain injury. In *KCNQ2* genes, the final outcomes are determined by the genotype and duration of seizure control. Early initiation of appropriate treatment and avoidance of secondary brain injury due to prolonged seizures are important. For mitochondrial disease, timely treatment with coenzyme Q10 and vitamins is important when energy is unbalanced due to fever or stress.

## 3. Common Appearance of Channelopathies and Metabolic Disease

In metabolic diseases, symptoms vary from being mild to severe. Common metabolic diseases in newborns include hypocalcemia (early- and late-onset), hypoglycemic encephalopathy, vitamin B_6_ deficiency, hyperthyroidism, and glucose transporter deficiency Seizures may frequently occur in metabolic disorders, such as non-ketotic hyperglycemia and Menkes disease. Similarly, seizures are a common symptom found in both channelopathies and neurometabolic disorders; these seizures can occur early in life within the first week. Other overlapping events of channelopathies and neurometabolic disorders include consciousness change due to seizure or antiepileptic intravenous medications, SB patterns in some channels and *KCNQ2* groups and, in some metabolic disorders, refractory seizure after antiepileptic medications (Table 1).

The common appearance of channelopathies and metabolic diseases includes seizures, SB EEG, and an initially unremarkable brain magnetic resonance imaging (MRI) (Table 1). Seizures can be mild to severe and may be refractory or manageable. Myoclonic seizures are predominant in neurometabolic diseases, such as those exhibited in non-ketotic hyperglycemia (NKH) or mitochondrial disease. In the study of Falsaperla R. et al., generalized tonic-clonic seizures were the most common type of pyridoxine-dependent seizures, with a usual EEG characterizing SB pattern [15]. These isolated seizures reveal pyridoxine-responsive and folinic acid-responsive seizures [16].

In channelopathies, seizures usually present as tonic (focal or general), such as those exhibited in *KCNQ2*, *SCN2A* and *SCN8A* genetic mutations (Fan et al., 2021). *KCNQ2*, *SCN2A*, and *SCN8A* genetic mutations have shown an initial ictal EEG recording that displayed a lower amplitude and fast activity [21,22,23]. *KCNQ2*, *SCN2A*, and *SCN8A* mutations often cause general and focal tonic seizures. They also demonstrated an EEG recording from unremarkable to severe SB EEG, while MRI scans ranged from unremarkable to severe (Table 1).

## 4. Differences between Neurological Channelopathies and Neurometabolic Disorders in Newborns

Differences exist between neurological channelopathies and neurometabolic disorders in newborns. However, precise differentiation is difficult, thereby requiring ample experience. The inheritance pattern in channelopathy is autosomal dominant, in which the mutations of patients can be de novo or inherited from one of the parents. In neurometabolic disorders, inheritance is often autosomal recessive, or maternal in mitochondrial genome mutations. Neurological channelopathies are rarely associated with other organ systems; in contrast, neurometabolic diseases are more associated with other multiple organ diseases. Many feeding and consciousness problems arise in metabolic diseases, but these problems may not be present in neurological channelopathies. Table 2 summarizes the differences between the two disorders.

### 4.1. Seizure Type

In *KCNQ2*-related neonatal developmental epileptic encephalopathy (DEE), brain MRI frequently shows bilateral or asymmetric hyperintensities in the basal ganglia, and sometimes in the thalamus, which may resolve over time [7,8,9,24]. Other common findings include small frontal lobes with increased adjacent extra-axial spaces, thin corpus callosum, and decreased posterior white matter volume [8,24]. However, specific seizure types, such as marked myoclonic seizures or distinctive electroencephalographic patterns, such as SB EEG patterns, epileptic syndrome, or early myoclonic encephalopathy, may suggest a specific metabolic disease [25]. In most cases, epilepsy secondary to inherited metabolic disorders presents with polymorphic clinical and electrographic features that are difficult to classify into precise epileptic syndromes.

### 4.2. Changes in Consciousness in Neurological Channelopathies and Neurometabolic Disorders in Newborns

Metabolic dysfunction is an important cause of neurological diseases, including neonatal epilepsy. Epilepsy rarely dominates the clinical presentation; rather, it is more frequently associated with other neurological symptoms, such as hypotonia and/or vigilance disturbances [25]. In neonatal channelopathy, the vigilance of newborns is not markedly different from that of newborns with neurometabolic disorders.

### 4.3. Amplitude-Integrated EEG (aEEG) and EEG Monitoring

EEG monitors are widely available in neonatal intensive care units (NICUs). Nonconvulsive seizures and nonconvulsive status epilepticus are common clinical observations in infants with encephalopathy [26,27,28]. The use of aEEG and EEG monitors can precisely define seizure semiology and a specific syndrome. Moreover, the combination of continuous EEG (cEEG) and aEEG data may allow a better distinction of seizures. The aEEG background is better at detecting channelopathy than detecting neurometabolic disorders. Low-voltage fast activity followed by recruiting spikes or theta rhythms arising mainly from the central regions of either hemisphere followed by focal spike-wave complexes and prolonged focal or diffuse postictal attenuation could be seen in channelopathies, such as *KCNQ2* and *SCN2A* channelopathy. Hypsarrhythmia on EEG, progression, and West syndrome are infrequently associated with channelopathies [24,29]. In metabolic disorders, the ictal period is more likely to have a delta-theta rhythm. During the interictal period, the EEG pattern was normal (Table 2).

### 4.4. Brain MRI

In neurological channelopathies, an MRI scan can usually be unremarkable or with hyperintensity in the basal ganglia. In one study involving *KCNQ2* mutations, there was progressive diffuse hypomyelination with marked thinning of the corpus callosum and prolongation in the lentiform nuclei that normalized a day later, which can be attributed primarily or secondarily to seizures [29]. An unremarkable image, hypomyelination (Pelizaeus-Merzbacher disease) to severe abnormalities in white matter (leukoencephalopathy), basal ganglia, thalamus, and brain stem (mitochondrial, organic aciduria) can be seen, depending on the progression of the disease [14]. Brain MRI of the *SUOX* gene mutation exhibited bilateral subcortical multi-cystic encephalomalacia involving bilateral parieto-occipital regions [30]. Maple syrup urine disease presents abnormal signals mainly involving the globus pallidus, thalamus, internal capsule, brainstem, and cerebellar white matter [30].

## 5. Syndrome Diagnosis

### 5.1. Developmental Epileptic Encephalopathies

DEE is age-specific and has diverse etiologies. Increasing evidence suggests that genetics play a pivotal role in pediatric DEE as well as in other severe neurological disorders [5,13,31]. Although the incidence of each disease is low, the combined incidence is inadequately estimated and remains unknown. DEEs are highly heterogeneous genetically, although genetic etiologies have been identified in only half of the cases, typically in the form of de novo dominant mutations. DEE-presenting infants include mutations in *KCNQ2*, *SCN1A*, *SCN8A*, and *SCN2A*. However, gene mutations may be ultra-rare and nonrecurrent in cases series [32]. The International League Against Epilepsy (ILAE) Task Force on Classification and Terminology [33] proposed to include electroencephalographic SB in the list of epileptic encephalopathies, i.e., those conditions that not only exhibit epileptic activity, but also epileptiform EEG abnormalities, which contribute to the progressive disturbance in cerebral function [34].

### 5.2. Early Infantile Epileptic Encephalopathy

Early infantile epileptic encephalopathy (EIEE) is a neurological disorder characterized by epileptic seizures, which affects newborns, usually within the first 3 months of life (usually within the first 10 days). EIEE with SB was first described in 1976 [35]. Since 1978, numerous articles have described an epileptic syndrome with either a neonatal onset or an onset in the first months of life, which is characterized by erratic, fragmentary myoclonus, massive myoclonus, partial seizures, late tonic spasms, and EEG signs such as SB. SBs indicate a major dysfunction of cortical networks, which might evolve into different patterns, as described in post-anoxic patients [36]. Infants have primarily tonic seizures, which cause stiffening of muscles (generally those in the back, legs, and arms), but they may also experience partial seizures and, rarely, myoclonic seizures (which cause jerks or twitches of the upper body, arms, or legs). The etiologies of EIEE include brain structural abnormalities and gene mutations, such as mutations in *STXBP* (9q34.1, encoding a syntaxin-binding protein), *SLC25A22* (11p15.5, encoding a mitochondrial glutamate carrier), *CDKL5* (Xp22, encoding a phosphorylated protein with protein kinase activity), *ARX* (Xp22.13, a homeobox-containing gene expressed during development), *SPTAN* (a family of filamentous cytoskeletal proteins), *PCDH19* (a member of the delta-2 protocadherin subclass of the cadherin superfamily), *KCNQ2*, and *SCN2A* genes [22,32,37]. Although their etiologies vary, their outcomes are generally unfavorable [38]. Genetic variants of EIEE have been associated with mutations in certain genes, such *ARX*, *CDKL5*, as *SL25A22* (encoding a mitochondrial glutamate carrier), and *STXBP1*. Episodes may occur more than a hundred times per day. Most infants with this disorder show underdeveloped cerebral hemispheres, in part or as a whole, or other structural anomalies. Some cases are caused by metabolic disorders or mutations in different genes [39].

*KCNQ2*-related EIEE should be distinguished from other early onset epileptic encephalopathies. *KCNQ2*-related NEE is also characterized by recurrent seizures, prominent interictal epileptiform discharges, and poor neurocognitive development. Although epileptic encephalopathies are often associated with structural brain defects or genetic metabolic disorders, pathogenic variants may also be involved in the development of epileptic encephalopathies, especially in the absence of clear genetic inheritance patterns or consanguinity [2]. EIEEs are genetically heterogeneous. Based on the genes in which pathogenic variants have been identified, the current classification of EIEE is as follows [2].

### 5.3. Early Myoclonic Encephalopathy (EME)

EME occurs in newborns at the onset of seizure before the first 2 weeks of life. The common types of seizure are prominent myoclonus, focal myoclonus, or focal. EEG typically exhibits an SB but may only be present during sleep. EEG SBs in newborns occur in a pattern of high-amplitude (>10 µV) discharges, usually consisting of slow waves with or without spikes, and alternate with periods of minimal low-amplitude (<10 µV) discharges [31,38,40]. The SB pattern is a characteristic signal in EEGs. According to the ILAE [33], early epileptic encephalopathy with SB consists of two distinct epileptic syndromes: EIEE and EME. The etiologies of EME include NKH, amino acids and organic acidopathies, urea cycle disorders, mitochondrial disorders, pyridoxine and pyridoxal-5-phosphate disorders, and sulfite oxidase deficiency. Prognosis is poor, and patients are often unresponsive to drugs, have severe intelligence disability, and are prone to early mortality.

## 6. Study Supporting the Diagnosis

Basic metabolic panel plus serum concentrations of calcium, magnesium, and phosphorus. Pyridoxine-dependent seizures are a rare genetic disorder of vitamin B_6_ metabolism caused by pathogenic variants in *ALDH7A1* (a member of subfamily 7 in the aldehyde dehydrogenase gene family), and is characterized by neonatal-onset seizures that are resistant to common anticonvulsants, but controlled by daily treatment with vitamin B_6_. Serum and urine alpha-AASA levels have been evaluated as biomarkers of this disorder. In this context, it should be noted that in a recent study, a de novo *KCNQ2* mutation (c.629G > A; p.Arg210His) was identified in a patient aged 7 years whose neonatal seizures showed a response to pyridoxine and who had a high plasma-to-CSF pyridoxal 5’-phosphate ratio, but no further proof of an inborn error of vitamin B_6_ metabolism [41]. Thyroid function tests, complete blood count, prothrombin time, activated partial thromboplastin time, and electrolyte imbalances, such as hypocalcemia and hypermagnesemia, are experienced in early- and late-onset seizures before the first week of the newborn. The etiologies include hypoparathyroidism, vitamin D deficiency, and DiGeorge syndrome.

### 6.1. Lumbar Puncture 

Cerebrospinal fluid is examined to rule out neonatal meningoencephalitis or occult blood, which can result in the differential diagnosis of meningitis, encephalitis, and glucose-transporter deficiency.

### 6.2. Brain MRI and/or CT Scan

It is recommended to distinguish neonatal seizures from structural lesions and intracranial hemorrhage. In channelopathies, early MRI after the first seizure is usually unremarkable. However, after recurrent, refractory seizures, the MRI can reveal brain atrophy or corpus callosum dysgenesis. The differences in the MRI scans of *SCN1A* and *PCDH19* mutations have been documented in Dravet syndrome with initial normal brain MRI scans that eventually progressed to cortical or cerebellar atrophy, cortical dysplasia, and temporal lobe abnormalities at 2–3 years. In addition, three of the five patients (60%) with *PCDH19* mutation revealed abnormal brain MRI, including mesial temporal sclerosis, multiple white matter nodules over subcortical and periventricular regions, and microcephaly. A case report consisted of five females with *PCDD19* mutation-related epilepsy who developed cortical malformations, including focal cortical dysplasia and PCDH19, which is thought to play a role in neuronal migration [11,42].

### 6.3. Electroencephalography, aEEG and EEG Monitoring

No specific EEG trait characterizes benign, familial neonatal seizures; the interictal EEG is commonly normal, occurring in 50–70% of infants. In the NICU, EEG monitoring is used extensively and contributes to early diagnosis, which is based on morphology and an aEEG picture. Nonconvulsive seizures and nonconvulsive status epilepticus, which are common in infants with encephalopathy and those with a high nonconvulsive seizure risk, do not exhibit clinically observable symptoms [27,28,43,44]. Hence, the rationale of aEEG and EEG monitoring in the NICU is to detect and verify diagnosis of seizure and, consequently, to provide adequate management [23].

The combination of cEEG and aEEG allow better detection of seizures, including clinical observation of seizure pattern and EEG seizure without clinical observation (electrographic seizures). Similarly, in a prospective study of 100 children with acute encephalopathy, Abend et al. noted electrographic seizures in 46%, electrographic status in 19%, and exclusively nonconvulsive seizures in 32% [45].

Use of video cEEG and aEEG data can define seizures precisely, and can differentiate the seizures from artifacts and environmental influence. The number of electrodes used in recording the neonatal EEG is reduced due to the smaller head circumference of a newborn. A modified standard 10–20 system is used with a single combined longitudinal and transverse montage. However, most clinical indications for an emergent EEG at this age do not require a high degree of spatial resolution, as the assessment of background activity is not affected by the reduced number of electrodes. The reduced montage has been shown to have a high sensitivity (96.8%) and 100% specificity when compared to a full 10–20 montage in detection of neonatal seizures.

The aEEG background classification remains inconsistent and is still being debated [46,47,48]. A multicenter study [44,48] comparing the simple system by al Naqeeb [49] versus the advanced scheme by Hellstrom-Westas et al. [26] showed that interobserver agreement was better when using the simple technique. Commercial aEEG systems have similar outputs. Neonatologists found better assessment using a simple aEEG system regardless of their expertise or the presence of seizures [46,48]. New aEEG tracings were generated using the NicoletOne Reader Software, which suggests the use of the classification as in the study. The aEEG can detect 46–76% of seizures [26,43,49]. Depending on the number of electrodes used and its placement, aEEG can be detected by conventional full-array EEG.

## 7. Drugs Treatment

**Figure 1:**
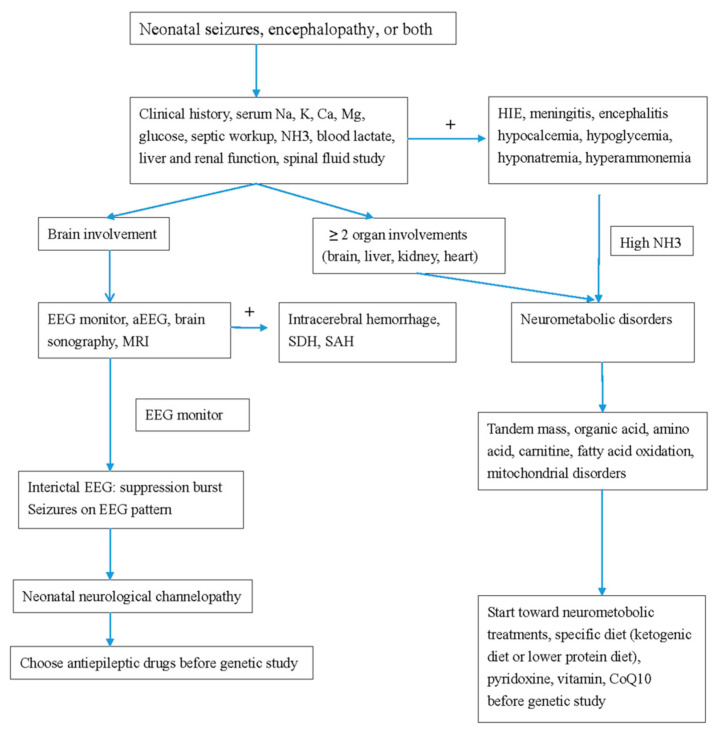
Flow chart of the study procedure in neonatal neurological channelopathy and neurometabolic disorders. MRI, magnetic resonance imaging; EEG, electroencephalography; aEEG, amplitude integrated EEG; SDH, subdural hemorrhage; SAH, subarachnoid hemorrhage; CoQ, coenzyme Q10.

**Figure 2:**
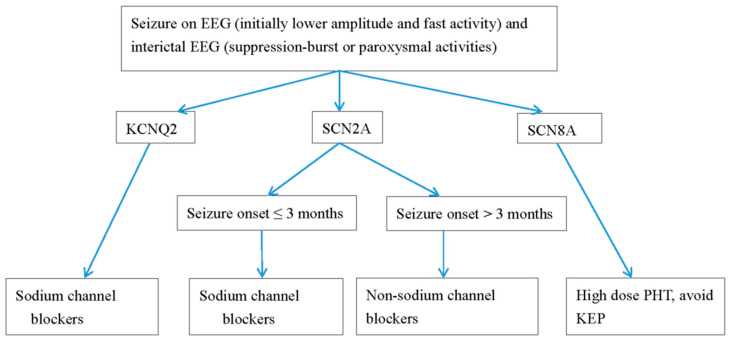
Antiepileptic drugs for neonatal seizures caused by channelopathy. PHT, phenytoin; OXC, oxcarbazepine; KEP, levetiracetam. EEG, electroencephalography.

Treatment plans depend on the etiology and can include supportive treatment for metabolic defects. In *KCNQ2* EE, refractory seizures can be the first choice of treatment with oxcarbamazepine [3,4,7,10,21,43,50,51,52]. Retigabine, a Kv7.2 opener, can reverse the conductance curve in vitro. However, the benefits and side effects in newborns still require a large number. In one systemic review investigating the effectiveness of drugs for *KCNQ2* seizures, sodium channel blockers including oxcarbazepine and phenytoin were most effective in 90% of patients, followed up by valproate and phenobarbital in up to 70%. The efficacies of levetiracetam and benzodiazepine were seen in less than 50% [53]. In *SCN2A*, *SCN8A*, and *KCNQ2* seizures with refractory seizures, sodium channel blockers are the standard drug choices. In a study conducted [4] (Table 2), all patients with *SCN2A* mutations and seizures were effectively treated with sodium channel blockers. A ketogenic diet and high-dose steroid treatment were also effective for *SCN2A* seizures. Mutations in *SCN2A* neonatal-to-infantile-onset patients younger than 3 months were missense mutations with gain of function, whereas mutations in patients with childhood-onset seizures were likely to be a loss of function or as a truncation mutation [4] (Table 2). Conversely, for children with autism spectrum disorder or intellectual disabilities with childhood-onset (≥3 months) seizures, non-sodium channels inhibiting antiepileptic drugs, including levetiracetam, benzodiazepines, and valproate, are the best treatment options [4]. For *SCN8A* drug-resistant seizures, high-dose Na-channel blockers should be considered [3,4,50,51] (Table 2). We demonstrate a flow chart of the study procedure in neonatal neurological channelopathy and neurometabolic disorders (Figure 1), and the anti-seizure drugs for treatment for neonatal channelopathy (Figure 2).

## 8. Conclusions

Early diagnosis and initiation of treatment before confirmation by genetic study, either as a channelopathy or neurometabolic disease, is critical for the long-term neurodevelopment of a child. Channelopathy should be considered if a patient presents with (1) predominantly general seizures, (2) unremarkable MRI findings, (3) normal aEEG background scores and (4) negative biochemical data, respectively. Conversely, neurometabolic disorders are considered if the presentation of symptoms includes (1) severe encephalopathy, as manifested by changes in consciousness, (2) positive cranial signs, (3) poor aEEG background, (4) myoclonic prominence and (5) the presence of focal lesions or brain atrophy on images. Therefore, a tentative diagnosis can be made before obtaining genetic results and initiating therapy.

## Figures and Tables

**Table 1:** Similar presentations in neonatal neurological channelopathy and neurometabolic disorders.

	Channelopathy	Neurometabolic Disorder
Age of seizure onset	Newborn (1–2 weeks after birth)	Newborn (1–2 weeks after birth)
Brain involvement	Positive	Positive
Seizure frequencies	+ to +++	+ to +++
EEG	Unremarkable to severe suppression-burst	Unremarkable to severe suppression-burst
MRI at age of seizure onset	Unremarkable	Unremarkable
MRI at follow up after first seizure	Brain atrophy (if not treated)	Brain atrophy (if not treated)

MRI, magnetic resonance imaging; EEG, electroencephalography; +++, daily; ++, weekly; +, less than weekly.

**Table 2:** Differences in the presentations and treatments in neonatal channelopathy and neurometabolic disorders.

	Channelopathy	Neurometabolic Disorder
Inheritance pattern	AD, de novo	AR or maternal inheritance
Organ involvement	Brain	Multiple organs
aEEG and EEG		
Background	Better	Worse
Seizure on EEG	Initially fast activity, followed up with deta-theta spikes	Delta-theta waves or spikes
Seizure type		
Tonic (general or focal)	+++	+
Myoclonic	+	+++
Initial MRI at first seizure	Unremarkable or mild ventriculomegaly	Varied
MRI at follow up	Brain atrophy	Variable depending on etiology
Treatment		
Antiepileptic drugs	Na channel blockers (PHT, OXC)	Pyridoxine, Na channel blockers, non-Na channel blockers
Avoid drug	KEP (SCN8A)	VPA (mitochondrial)
Diet	Normal to ketogenic diet if uncontrolled seizures	Variable depending on etiology (ketogenic diet, lower protein diet, pyridoxine)
Outcomes in neurodevelopment	Unremarkable to severe	Moderate to severe

AD, autosomal dominant; AR, autosomal recessive; PHT, phenytoin; OXC, oxcarbazepine; VPA, valproic acid; KEP, levetiracetam; MRI, magnetic resonance imaging. EEG, electroencephalography; +++, daily; +, less than weekly.

## Data Availability

Not applicable.
